# Supplementary material for: Characterization of a Human Neuronal Culture System for the Study of Cofilin–Actin Rod Pathology
Source: Biomedicines. 2023 Oct 31;11(11):2942. doi: 10.3390/biomedicines11112942 (PMC10669520; doi:10.3390/biomedicines11112942)
Supplement: Supplementary file 1 [file biomedicines-11-02942-s001.zip › biomedicines-2670601-supplementary.pdf]

Supplementary Material

Characterization of a Human Neuronal Culture System for the Study of Cofilin–Actin Rod Pathology

Lubna H. Tahtamouni [1,2,†], Sydney A. Alderfer [3,†,‡], Thomas B. Kuhn [2], Laurie S. Minamide [2], Soham Chanda [2], Michael R. Ruff [4] and James R. Bamburg [2,*]

[1] Department of Biology and Biotechnology, Faculty of Science, The Hashemite University, Zarqa 13133, Jordan; lubnatahtamuni@hu.edu.jo
[2] Department of Biochemistry and Molecular Biology, Colorado State University, Fort Collins, CO 80523, USA; tom.kuhn@colostate.edu (T.B.K.); laurie.minamide@colostate.edu (L.S.M.); soham.chanda@colostate.edu (S.C.)
[3] Department of Chemical and Biological Engineering and School of Biomedical Engineering, Colorado State University, Fort Collins, CO 80523, USA; sydney.alderfer@gmail.com
[4] Creative Bio-Peptides, Inc., 10319 Glen Road, Suite 100, Potomac, MD *20854*, USA; mruff@creativebiopeptides.com
* Correspondence: james.bamburg@colostate.edu
† These authors contributed equally to this work.
‡ Current address: Buck Institute for Research on Aging, 8001 Redwood Blvd., Novato, CA 94945, USA.

Supplementary Methods

SH-SY5Y cells: Human SH-SY5Y neuroblastoma cells were obtained from American Type Culture Collection (ATCC CRL-226) and maintained in an undifferentiated phenotype by growth on tissue culture plastic dishes in HG-DMEM supplemented with 2 mM L-glutamine (ThermoFisher), 10% HyClone fetal bovine serum (VWR) and 100 U/mL penicillin/streptomycin (VWR) at 37°C as a growth medium. SH-SY5Y cells were neuronally differentiated using a previously described protocol [1] with a minor modification. Briefly, cells were plated at 10,000/ml onto 12 mm glass coverslips coated with extracellular matrix (MaxGel ECM, Sigma) in 24 well plates and subjected to three different differentiation media thereby sequentially decreasing FBS concentration from 10% to 2.5% to 1% to serum-free in the presence of 10 μM retinoic acid over a 12-day period. Final medium change was into homemade neurobasal medium (hNB) with 175 μM L-cysteine also containing 1x B27 (ThermoFisher), 20 mM KCl, 2 mM L-glutamine, 100 U/ml penicillin/100 μg/ml streptomycin, 2 mM dibutyryl-cAMP, 10 μM retinoic acid, and 50 ng/ml brain-derived neurotrophic factor.

Human embryonic stem (hES) cells: Cultures of neurons derived from H1 hES cells were established using a slight modification of the original protocol [2,3] following the timeline shown in **Figure S5B.**

Supplementary Figures

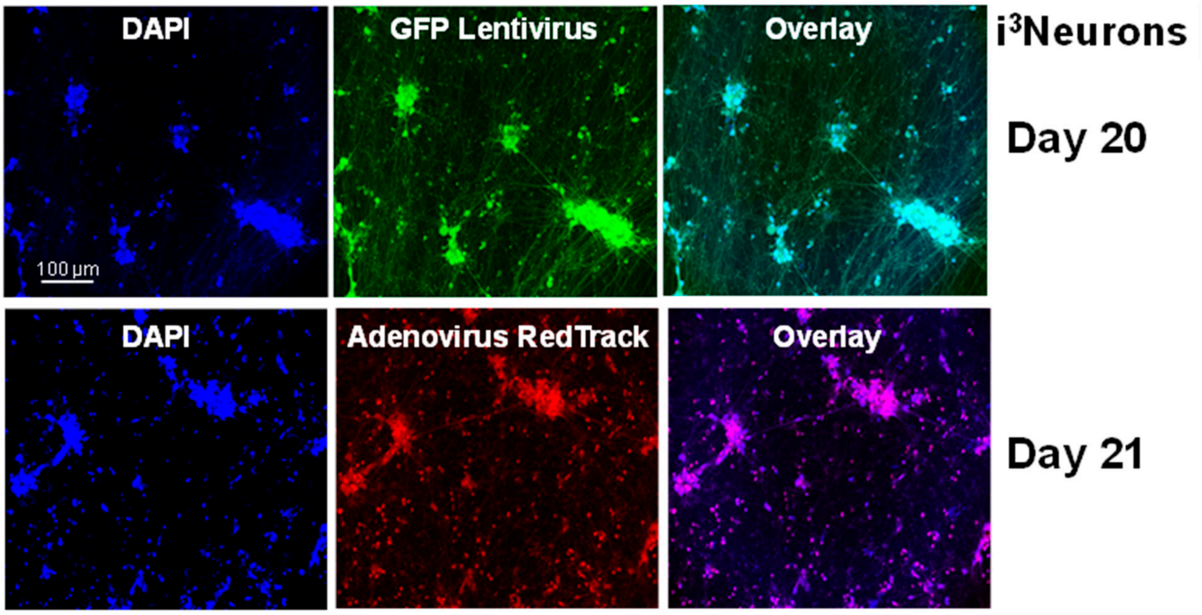

Figure S1. **i**^{3}**Neurons are efficiently infected by both lentivirus and adenovirus.** Top panels show i^{3}Neurons infected on day 10 with lentivirus for GFP expression and fixed on day 20. Greater than 99% of nuclei (DAPI label) overlap with GFP. Lower panels show i^{3}Neurons infected on day 17 with 30 moi of adenovirus expressing mRFP (AdRT). Cells were fixed on day 21. As with lentivirus, greater than 99% of nuclei (DAPI) and mRFP overlap.

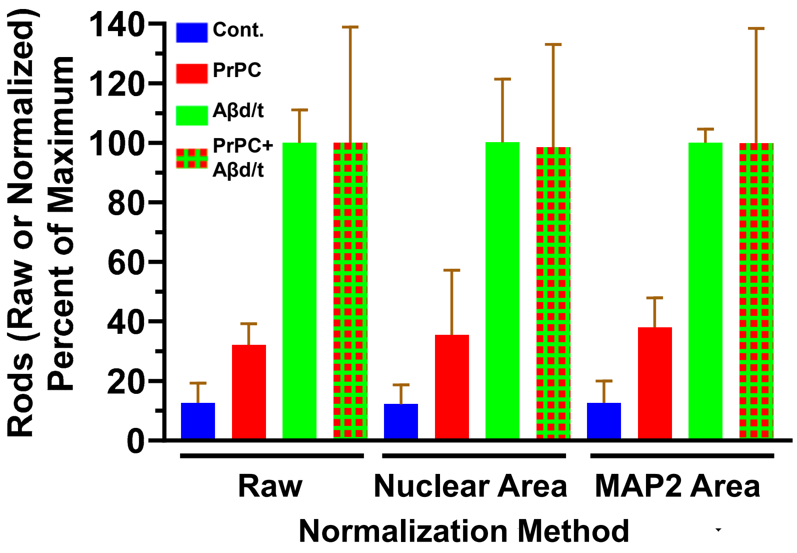

Figure S2. **Normalization of rod numbers to either total area of nuclei (DAPI staining) or MAP2 (immunolabel) gives nearly identical results regardless of the intensity of the rod response in day 55 i^{3}Neurons.** To determine if a decline in MAP2 immunolabeling due to rod-induced cytoskeletal disruption alters rod normalization after counting raw rod numbers, the rod counts from intact full 7x7 image arrays from cultures (4-6) containing untreated (control), Aβd/t-treated, PrP^C-expressing, and Aβd/t treated PrP^C expressing were first adjusted to the percentage of the average maximum response for the treated culture plate (Raw) and then normalized by dividing by either nuclear area (DAPI staining) or MAP2 immunolabeled area. Normalization to either nuclear area or MAP2 immunolabeled area show no difference and are also nearly identical to the raw image counts when adjusted to their percentage of the rods in the wells yielding the maximum response, provided full and complete 7x7 image arrays are used. However, not every coverslip has a region where a full array can be obtained. Normalization to MAP2 area was used for all quantitative rod data from cultures day 35 and older to correct for lost regions and readjusted to the area of the full 7x7 array. NF-H was used similarly for cultures younger than 27 days. Error bars are standard deviations and show rod numbers quantified from PrP^C-expressing i^{3}Neurons show more variability than rods treated with Aβd/t alone.

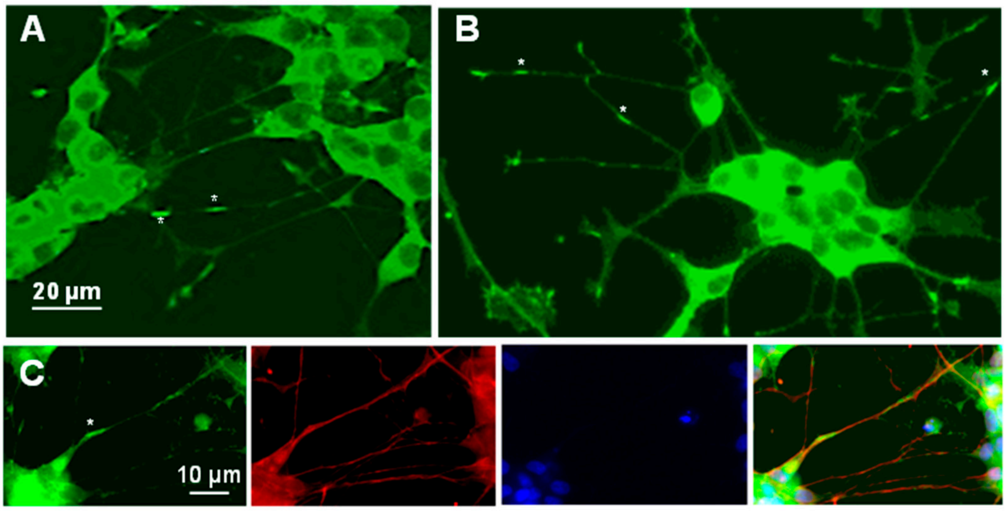

Figure S3. **Cofilactin rods in SH-SY5Y cells in response to energy depletion and stimulators of the PrP^C-dependent pathway.** Energy depletion with (**A**) antimycin A or (**B**) sodium azide/2-deoxyglucose causes formation of rod-shaped cofilin aggregates (asterisks) in processes of differentiated SY5Y neurons. (**C**) Adenoviral (100 moi)-mediated expression of PrP^C in the SH-SY5Y cells 3 days prior to treating 18 hr with gp120$_{MN}$ (500 nM dual tropic). Cofilin immunolabeling (green, *rod), neurofilament-H (red) and DAPI (blue) and shown in overlay on right. Expression of PrP^C alone also induced cofilin rods in these cells, suggesting other necessary components of the PrP^C/NOX pathway are intact. However, a considerable number of other irregular cofilin/actin structures forming within neurites added ambiguity to rod analysis. Coupled with the short life span of cultures and the heterogeneity previously found in the electrophysiological properties of SH-SY5Y cells [4], they were eliminated from further consideration as a human neuronal model for rod studies.

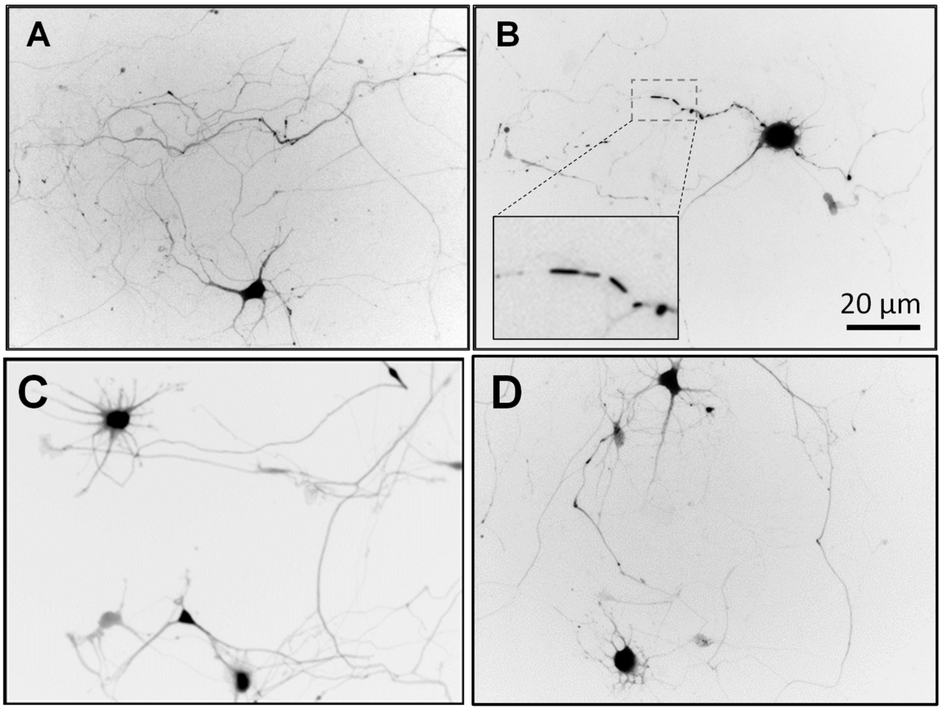

Figure S4. Glutamatergic neurons (28 day) derived from human ES cells cultured with glia and expressing a fluorescent rod reporter form rods in response to glutamate but not Aβd/t. Inverted fluorescence images of glutamatergic neurons derived from human ES cells by NGN2 transfection [2,3]. **See timeline for hES cells in Figure S5B.** Because of the presence of glia, immunolabeling for cofilin gives a high background from which visualization of rods becomes difficult. To determine if hES-derived neurons might be used for rod assays in live cultures, neurons in co-culture with rodent glia for 14 days were infected with a lentivirus for expressing a cofilactin rod reporter, cofilinR21Q-mRFP [5]. Expression was driven by the neuronal-specific synapsin promoter. Neurons were infected with adenovirus for expressing cofilinR21Q-mRFP on day 24 and were fixed and imaged at day 28. (**A**) untreated control. (**B**) Treated with 150 µM glutamate for 2 hr before fixation. Inset shows magnified region of cofilactin rods. Qualitative examination of cultures showed over 80% of cells expressing cofilinR21Q-mRFP formed rods following glutamate treatment. (**C, D**) Treatment with Aβd/t 24 hr prior to fixation failed to induce rods above those in untreated neurons (random sample chosen).

A. Timeline for making neurons from human WTC-11 iPSCs

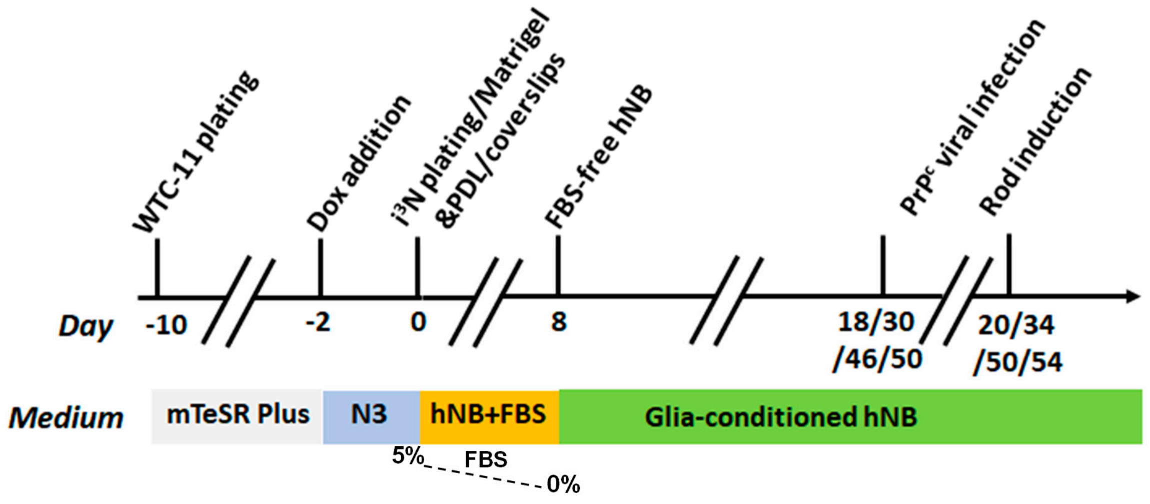

B. Timeline for making neurons from hES cells

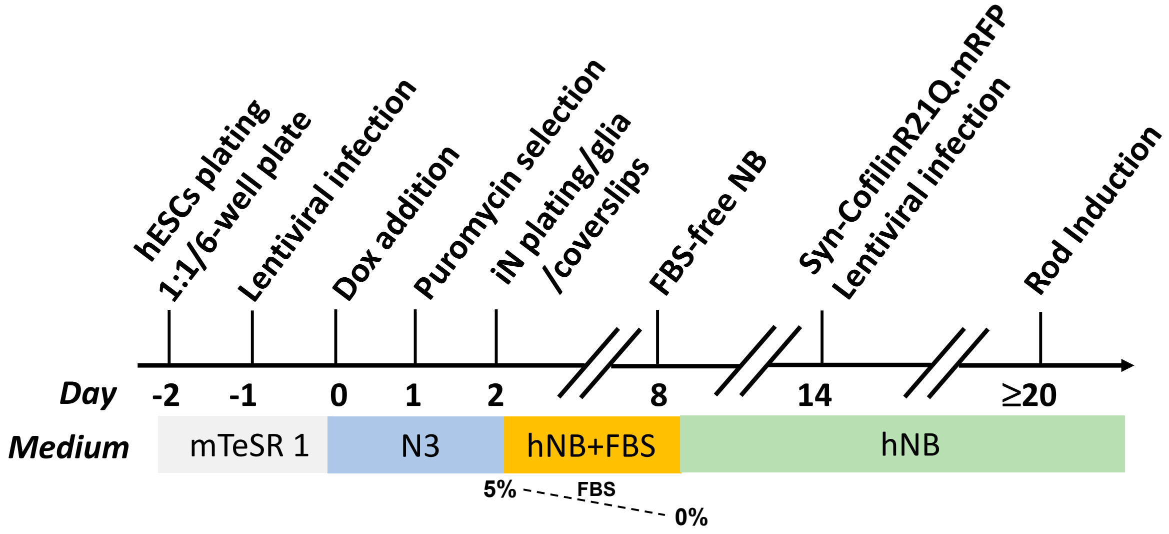

Figure S5. **Timelines for deriving neurons from WTC-11 iPSC and hES cells.** (**A**) WTC-11 human iPSCs were converted to neurons using an integrated, isogenic, doxycycline-inducible NGN2 gene. After plating on coverslips on Day 0, the FBS was reduced by 50% each day from Day 0 to 8 at which point glial-conditioned medium was used. Adenoviral expression of PrP^C was performed 3 to 4 days in advance of treatment with rod inducers. (**B**) Human embryonic stem cells (line H1) were infected with lentiviruses for doxycycline regulated NGN2 and GFP expression on day -1. Puromycin selection was on day 1 and serum reduction was done over 6 days after plating cells on glia. Cells were infected on day 14 with lentivirus for synapsin promotor-driven expression of cofilinR21Q-mRFP and rod induction was done days 24 to 28.

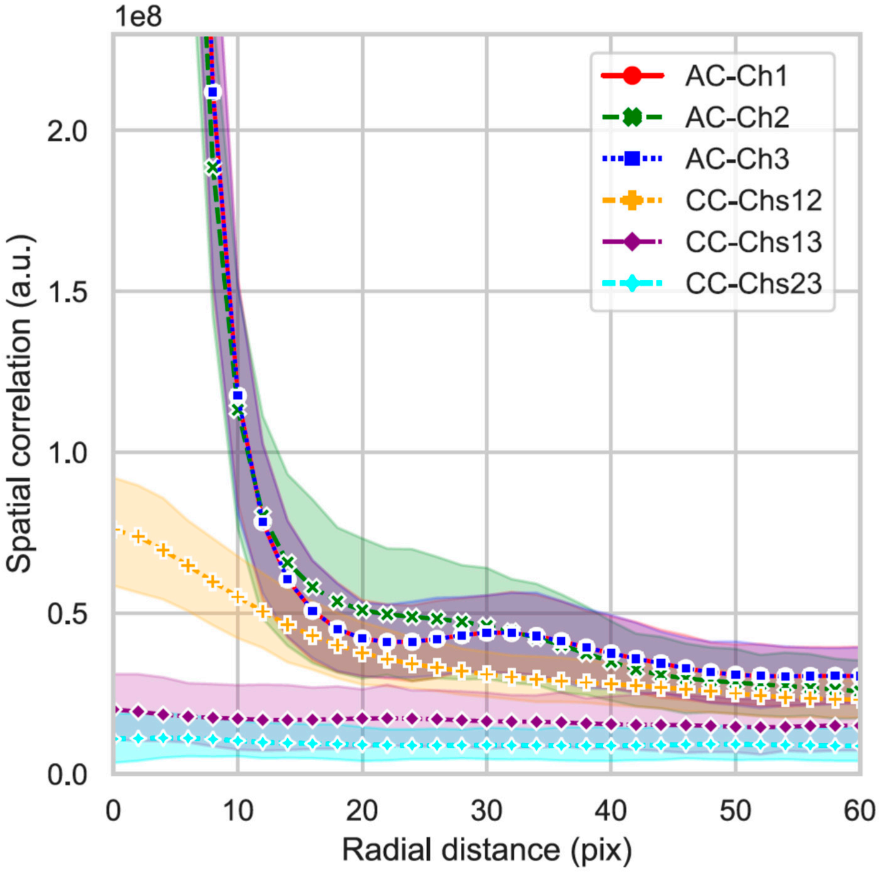

Figure S6. Spatial auto- and cross-correlation between PSD95 and VGLUT puncta. To determine if the contact/overlap between VGLUT (Ch1) and PSD95 (Ch2) puncta is due to a random association, the two images from 12 fields (data in **Table S1**) were analyzed for pixel overlap followed by a spatial cross-correlation (CC) and auto-correlation (AC) between signals as well as between each image and a mirrored and flipped image of PSD95 (Ch3) [6]. One overlayed image is moved two pixels at a time in each direction surrounding its paired image to a radial distance of 100 pixels, only 60 of which are shown on the plot. The auto-correlation (AC) shows the complete overlay of each image with itself whereas cross-correlation (CC) shows that the maximum overlap (CC-Chs12) between VGLUT and PSD95 occurs at a zero-pixel adjustment, showing that they are maximal in the original images. By the radial movement of ~25 pixels, no correlation exists, and the slope is about equal to that of the non-correlated flipped PSD95 (Ch3) image with both its unflipped counterpart (Chs23) and VGLUT (Chs13). Thus, the contacts observed between PSD95 and VGLUT are not random. The peak in the auto-correlation images at 32 pixels represents an approximate size of puncta. Shaded areas show the 95% confidence level from the combined curves.

Image Ref.	PSD95 VGLUT Contacts	PSD95 Puncta Number	% PSD95 overlap w VGLUT	PSD95 % Area	PSD95 Puncta Pixel Size	VGLUT Puncta Number	VGLUT % Area	VGLUT Puncta Pixel Size	MAP2 Area μm2	MAP2 % Area	PSD95/MAP2 Area Ratio
CS1-17	120.00	714.00	16.80	1.77	22.70	517.00	1.64	21.70	556.00	11.15	1.28
CS2-13	135.00	551.00	24.50	2.64	23.10	563.00	2.05	22.20	729.00	14.60	0.76
CS2-14	113.00	689.00	16.40	1.98	23.00	536.00	1.56	22.00	890.00	17.85	0.77
CS3-1	188.00	731.00	25.70	1.92	22.10	1143.00	2.79	21.90	1020.00	20.50	0.72
CS3-16	153.00	413.00	37.00	2.23	23.80	709.00	2.08	23.30	690.00	13.80	0.60
CS1-10	123.00	998.00	12.30	2.41	22.70	631.00	1.40	20.70	777.00	15.60	1.28
CS1-7	130.00	999.00	13.00	2.39	22.40	792.00	1.92	21.00	614.00	12.30	1.63
CS1-20	102.00	1027.00	9.93	2.50	22.20	369.00	0.82	20.40	680.00	13.60	1.51
CS2-16	117.00	613.00	19.10	1.57	23.30	741.00	2.58	22.60	657.00	13.20	0.93
CS2-17	120.00	623.00	19.30	2.10	23.20	617.00	1.74	22.20	1135.00	22.80	0.55
CS3-10	109.00	552.00	19.70	1.81	25.30	318.00	1.65	26.00	440.00	8.80	1.25
CS3-15	95.00	578.00	16.40	1.54	23.10	415.00	1.17	22.40	524.00	10.50	1.10
ave	125.42	707.33	19.18	2.07	23.08	612.58	1.78	22.20	726.00	14.60	1.03
Std. Dev (99%)	±17.7	±142	±5.2	±0.261	±0.61	±158	±0.39	±1.03	±144.9	±2.92	±0.26

Table S1. Imaging data from spinning disc confocal microscope used to quantify the PSD95/VGLUT interactions and MAP2 areas. Data are from 12 fields from three cultures (CS1-3) with field numbers of images selected at random from 25 confocal captures per culture. Each field is 4,985 μm^2 and contains 2,150,400 pixels (431.4 pixels per μm^2). Projection images of a 3 μm stack (0.1 μm/step) were deconvolved (Slidebook 2023.1) and thresholded to remove weakly fluorescent particles in each channel and then manually scored for contact between postsynaptic (PSD95) and presynaptic (VGLUT) puncta. All puncta between 5 and 50 pixels in size were selected from color separated images (Image J) as these covered the common size range of pre- and post-synaptic areas (0.2 to 0.4 μm diameter or ~13 to 50 pixels). Each field was analyzed for MAP2 (dendrite) area, which averaged 14.6%. Bottom row shows standard deviations (sd) at 99% confidence for the averages in the row above.

Supplementary References

1. Shipley, M.M.; Mangold, C.A.; Szpara, M.L. Differentiation of the SH-SY5Y Human Neuroblastoma Cell Line. *J. Vis. Exp. JoVE* **2016**, 53193, doi:10.3791/53193.
2. Zhang, Y.; Pak, C.; Han, Y.; Ahlenius, H.; Zhang, Z.; Chanda, S.; Marro, S.; Patzke, C.; Acuna, C.; Covy, J.; et al. Rapid Single-Step Induction of Functional Neurons from Human Pluripotent Stem Cells. *Neuron* **2013**, *78*, 785–798, doi:10.1016/j.neuron.2013.05.029.
3. Chanda, S.; Ang, C.E.; Lee, Q.Y.; Ghebrial, M.; Haag, D.; Shibuya, Y.; Wernig, M.; Südhof, T.C. Direct Reprogramming of Human Neurons Identifies MARCKSL1 as a Pathogenic Mediator of Valproic Acid-Induced Teratogenicity. *Cell Stem Cell* **2019**, *25*, 103-119.e6, doi:10.1016/j.stem.2019.04.021.
4. Santillo, S.; Schiano Moriello, A.; Di Maio, V. Electrophysiological Variability in the SH-SY5Y Cellular Line. *Gen. Physiol. Biophys.* **2014**, *33*, 121–129, doi:10.4149/gpb_2013071.
5. Mi, J.; Shaw, A.E.; Pak, C.W.; Walsh, K.P.; Minamide, L.S.; Bernstein, B.W.; Kuhn, T.B.; Bamburg, J.R. A Genetically Encoded Reporter for Real-Time Imaging of Cofilin-Actin Rods in Living Neurons. *PloS One* **2013**, *8*, e83609, doi:10.1371/journal.pone.0083609.
6. Saxton, M.N.; Morisaki, T.; Krapf, D.; Kimura, H.; Stasevich, T.J. Live-Cell Imaging Uncovers the Relationship between Histone Acetylation, Transcription Initiation, and Nucleosome Mobility. *Sci. Adv.* **2023**, *9*, eadh4819, doi:10.1126/sciadv.adh4819.
